# SERSNet: Surface-Enhanced Raman Spectroscopy Based Biomolecule Detection Using Deep Neural Network

**DOI:** 10.3390/bios11120490

**Published:** 2021-11-30

**Authors:** Seongyong Park, Jaeseok Lee, Shujaat Khan, Abdul Wahab, Minseok Kim

**Affiliations:** 1 Department of Bio and Brain Engineering, Korea Advanced Institute of Science and Technology (KAIST), Daejeon 34141, Korea; sypark0215@kaist.ac.kr (S.P.); shujaat@kaist.ac.kr (S.K.); 2 Department of Mechanical System Engineering, Kumoh National Institute of Technology, Gumi 39177, Korea; leelion7027@kumoh.ac.kr; 3 Department of Aeronautics, Mechanical and Electronic Convergence Engineering, Kumoh National Institute of Technology, Gumi 39177, Korea; 4 Department of Mathematics, Nazarbayev University, Nur-Sultan 010000, Kazakhstan; abdul.wahab@nu.edu.kz

**Keywords:** Surface Enhanced Raman Spectroscopy, molecule detection, machine learning, deep learning

## Abstract

Surface-Enhanced Raman Spectroscopy (SERS)-based biomolecule detection has been a challenge due to large variations in signal intensity, spectral profile, and nonlinearity. Recent advances in machine learning offer great opportunities to address these issues. However, well-documented procedures for model development and evaluation, as well as benchmark datasets, are lacking. Towards this end, we provide the SERS spectral benchmark dataset of Rhodamine 6G (*R*6*G*) for a molecule detection task and evaluate the classification performance of several machine learning models. We also perform a comparative study to find the best combination between the preprocessing methods and the machine learning models. Our best model, coined as the SERSNet, robustly identifies *R*6*G* molecule with excellent independent test performance. In particular, SERSNet shows 95.9% balanced accuracy for the cross-batch testing task.

## 1. Introduction

Surface-Enhanced Raman Spectroscopy (SERS) is a commonly used sensing technique that shares the advantages of conventional Raman spectroscopy, such as easy sample preparation, molecular fingerprinting, and low signal attenuation by solvents, while improving sensitivity. Specifically, the surface of the SERS device, which is often coated with metal nanoparticles, induces surface plasmon resonance localized on the metal surface to amplify the Raman scattering signal of the target molecule by up to $10^{8}$ or more [1]. Therefore, the SERS provides greater system design flexibility than Raman spectroscopy, making it suitable for portable applications such as detection of pathogen [2], water pollutant [3,4], and counterfeit [5], etc. Despite these successes, it is difficult to identify meaningful patterns in the SERS measurements and this often requires sophisticated signal processing techniques due to the inherent fluctuations and nonlinearities of signals originating from interactions between target molecules and the surface of the SERS device.

The recent advancement of machine learning (ML) provides opportunities to resolve these problems. Machine learning-based improvement of the biosensors is also reported recently. For example, Meyer et al. [6] proposed an SVM-based classification model to improve the DNA biosensor. Hassoun et al. [7] proposed an SVM-based classification model to classify three cell types. Singh et al. [8] reviewed recent advancements in electrochemical biosensors and the application of machine learning in these biosensing applications including the SERS biosensors. In addition, some of the recent studies have also demonstrated successful biosensing applications in response to the COVID-19 pandemic, such as in the detection of SARS-CoV-2 related proteins or in the detection of the virus itself [9,10]. These efforts provide examples of the successful application of machine learning techniques in biosensing. Other studies have reported successful applications of the ML models on the SERS measurements, see, for example, in [11,12,13,14,15,16,17,18]. For an instance, Amjad et al. [11] developed a *random forest* (RF) classifier to identify the origin of milk from four different species. The test accuracy of the trained random forest (RF) classifier was reported to be 93.97%. Dies et al. [13] reported a new SERS substrate assembly method and the proposed *support vector machine* (SVM)-based illicit drug detection model. Their reported accuracy of the identification of cocaine was 100%. Kim et al. [15] reported a paper-based SERS device for diagnosing prenatal disease in women. They used *principal component analysis SVM* (PCA-SVM) as a classifier to detect abnormal status from amniotic fluids. Their reported accuracy of the device was above 93%. Weng et al. [19] proposed some deep learning (DL) models for drug recognition in urine using *fully connected neural network* (FCNN), and *convolution neural network* (CNN). They have compared the accuracy of their model with conventional ML models such as *random forest* (RF), K-nearest neighbor (KNN)-based classifier, and SVM. Their reported best test accuracy was 98.05%. Leong et al. [20] have proposed an SERS-based taster which can recognize wine flavors. They have combined the so-called *SERS taster* with the SVM model to detect molecules for different flavors such as menthol, linalool, and limonene. Ciloglu et al. [21] proposed an SERS-based pathogen detection using the DNN. They classify *multi-drug resistant staphylococcus aureus* (MRSA) to *methicillin-sensitive staphylococcus aureus*. We refer to the review article [18] that summarizes machine learning and deep learning applications for the SERS biosensors including food, forensics, pathogen detection, medical diagnosis, and chemometric sensors. More general discussion about the application of machine learning and deep learning for Raman spectroscopy can be found in [22]. A summary of the research conducted on machine learning-based SERS biosensor is presented in Table 1.

The aforementioned studies suggest that the ML models can effectively solve the specific molecule detection problem using the SERS measurements. Unfortunately, relevant datasets and models are generally not furnished, so it is impossible to benchmark if one wants to improve the model performance against existing methods. Moreover, there is a lack of consensus in the preparation of the dataset and evaluation of the models, so the reported performance of the existing models could be questionable. Furthermore, there is limited discussion about the relationship between preprocessing of the SERS dataset and the performance of the ML and DL models. Therefore, it is often difficult to choose appropriate techniques for specific models to perform new molecule detection tasks. Although a recent study discusses a statistical approach for background removal for the SERS dataset, it is specialized in flow-based SERS sensor combined with the LC-MS [23] and did not provide an in-depth discussion about the relationship between ML/DL models and preprocessing techniques.

Detection of biomolecules by the SERS measurements has been extensively studied, including nucleotides, nucleic acids, amino acids, peptides, and proteins [24]. However, the acquired SERS signal is difficult to analyze due to the inherent variability of each SERS device fabrication method and the nonlinearity of the signal. Towards this end, many studies have focused on fabricating reproducible devices to reduce measurement variabilities, see, for instance, in [25,26,27]. Unfortunately, little effort has been devoted to developing methods based on signal processing and machine learning.

Several applications of machine learning have been reported in the fields of the SERS signal acquisition and data analysis [18,19]. However, there was not enough discussion about the performance of the machine learning models according to the SERS preprocessing methods and the reproducibility according to the *batch-effect*. To solve this problem, different normalization methods, such as Power Spectrum density Normalization (*PSN*) and feature-specific Batch Normalization (*BN*), were considered in this study to prepare a benchmark for the performance evaluation of various machine learning models. In addition, two independent experimental batches were constructed to conduct training and independent evaluation for examining the reproducibility of the trained models. The combinations of optimal model and preprocessing techniques for R6G molecule detection were derived by examining the variations in model performance between batches through the independent test set evaluation.

The R6G is a widely used molecule for the characterization of biosensors. It has been extensively used for molecule tagging in several bio-applications. For example, Chen et al. [28] proposed SERS-based surface-corrugated nanopillars for biomolecular detection of colorectal cancer. In their experiment, they used R6G molecule to characterize the sensing mechanism of their SERS device, which utilized quenching of fluorescence molecule Cy5. Tzeng et al. [29] also used the R6G as a control molecule for their adenine detection SERS sensor. Similarly, Vikulina et al. [30] verified the analytical performance of porous Au micro shells for detection of Rhodamine B. Sung et al. [31] used the R6G to characterize the performance of SERS substrate. These examples illustrate the importance of the R6G detection task in biosensing applications.

In this study, we used R6G as a proof of a concept (POC) molecule for the basic SERS + ML/DL biosensing concept which can be applicable to a wide range of applications of the SERS-based sensing techniques. Specifically, we propose the SERS-based molecule detection model using a deep neural network, coined as the *SERSNet*. To train the proposed SERSNet, we first design a new benchmark dataset for molecule detection tasks in the SERS measurements. We use Rhodamine 6G (R6G) as our target molecule as it is a well-characterized and widely used molecule in the SERS-based biomolecule detection applications such as protein detection [32]. Then, we conducted an extensive explanatory data analysis (EDA) on the SERS dataset to provide an insight into the relationship between different preprocessing techniques and the performance of different machine learning methods for the SERS-based molecule detection tasks. The performance of the trained model is evaluated on an independently measured SERS spectra.

This article is organized as follows. The material and method used in this study are discussed in Section 2, followed by a detailed analysis of experimental results in Section 3. Finally, the conclusions are drawn in Section 4.

## 2. Materials and Methods

**Figure 1:**
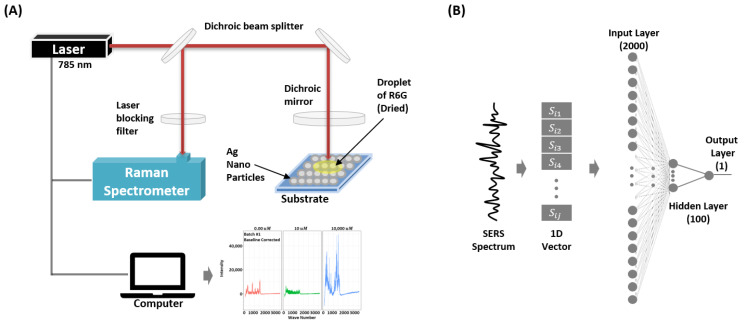
Configuration of the proposed surface-enhanced Raman spectroscopy-based R6G molecule detection using deep neural network SERSNet. (**A**) Experimental setup for the SERS measurements. (**B**) Proposed SERSNet architecture.

In this section, we provide a detailed description of the proposed SERS-based molecule detection framework. Figure 1 presents the configuration of the proposed method.

### 2.1. SERS Measurements

In this study, we use Rhodamine 6G (R6G) as our target molecule. The R6G was purchased from Sigma Aldrich (Seoul, South Korea) and the molecule is prepared in deionized water. We use commercially available SERS substrates (Kwanglim Precision Co., Ltd., Daegu, South Korea) for the measurements. The wavelength of the Raman spectrometer (NS200, Nanosystems Co., Ltd., Daejeon, South Korea) is 785 nm, and the laser power and exposure time are fixed at 200 mW and 500 ms, respectively. To acquire the SERS spectra, we drop a 2.5 μL sample on the SERS substrate and dry it at room temperature (27 °C). To minimize signal degradation, each SERS measurement is recorded with 10 s intervals. Each measurement sample $S\in R^{1\times 2000}$ (SERs spectrum) consists of 2000 wave-numbers (attributes). Figure 1 shows the experimental setup used for the measurement of the SERS dataset.

For each concentration, the SERS measurments are acquired using a separate substrate. We perform two consecutive experiments, named as *bacth1* and *batch2*. In each batch, we have 500 negative $S_{N}\in R^{500\times 2000}$ and 1500 positive $S_{P}\in R^{1500\times 2000}$ samples. The concentration of ≥0.01 μM is used as the threshold for positive (detection) which is in accordance to the reported limit of detection of the R6G molecule [33]. In particular, we acquire 5 concentrations data. In *batch1*, we measure 0 μM, 10 μM, and 10,000 μM. In *batch2*, 0.01 μM, 0.1 μM, and 100 μM are measured. Complete description of the sample distribution is provided in Table 2.

### 2.2. Preprocessing

In machine learning-based model designing, data preprocessing is one of the crucial steps. Towards this end, we use two normalization techniques. The normalization is the removal of sources of systematic variation between sample profiles to ensure that the spectra are comparable across related sample sets [34]. In particular, we consider *power spectrum density normalization* (PSN) and *feature-specific batch normalization* (BN). The PSN for *j*-th wavenumber of *i*-th sample $S_{i,j}$ is defined as

(1) $${S\mathrm{psn}}_{i,j}=\frac{S_{i,j}}{\sum S_{i}},$$

where Spsn is the power spectrum normalized signal and $\sum S_{i}$ is the sum of all intensity values for a sample $S_{i,j}$. Similarly, the BN for *j*-th wavenumber of *i*-th sample $S_{i,j}$ is defined as

(2) $$S{\mathrm{bn}}_{i,j}=\frac{S_{i,j}-\mu_{S_{j}}}{\sigma_{S_{j}}},$$

where Sbn is the batch-normalized signal, and $\mu_{S_{j}}$ and $\sigma_{S_{j}}$ are, respectively, the mean and standard deviation for all samples within a batch.

### 2.3. Model Configurations

Figure 1B shows the architecture of the proposed SERSNet. The proposed SERSNet model is based on a multi-layer perceptron (MLP) neural network. The architecture of the proposed MLP network consists of a single input layer of length 2000, a hidden layer with 100 neurons, and an output layer with only one neuron providing binary output to detect signal. For all neurons, *rectified linear unit* (ReLU) activation is used, with an exception of the output layer where *logistic* activation is used.

### 2.4. Model Training

The SERSNet is trained using 80% data from a single batch. For data splitting, we use stratified splitting method using *train test split* function in *scikit-learn* [35] package. After model training, the remaining 20% is used for validation, and a model with greater than 90% balanced accuracy is used for testing. Later, the trained model is used for the performance evaluation on the independent dataset (obtained from a different batch). The model is trained to minimize *log-loss function* using *Adam* optimizer. The model is implemented using the *scikit-learn* package with default settings on Python 3.

### 2.5. Performance Evaluation

It is worthwhile mentioning that conventional accuracy is not suitable to quantify the true performance due to the imbalanced nature of our dataset. Thus, we use the *balanced accuracy* (BACC) as our primary performance metric supplemented with the other metrics such as sensitivity, specificity, F1 score, *Matthews correlation coefficient* (MCC), and *Youden’s* index. To analyze threshold-independent performances, we used the area under the curve (AUC) for receiver operating characteristic (ROC) and precision–recall (PR) curves.

## 3. Results and Discussion

We qualitatively evaluate the identification difficulty of the measured SERS data through PCA using different preprocessing techniques in Section 3.1. Later, the results of the proposed model are discussed in Section 3.2. Finally, the results are compared with other state-of-the-art machine learning techniques in Section 3.3.

### 3.1. Exploratory Data Analysis

**Figure 2:**
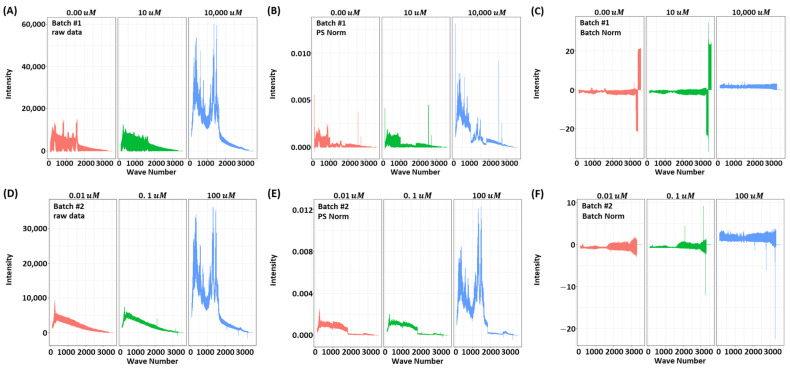
Visualization of the R6G SERS spectrum for *RAW*, power spectrum density normalization (PSN), and feature-specific batch normalization (BN) methods. (**A**–**C**): Batch1 RAW, PSN and BN prepressed datasets, (**D**–**F**): Batch2 RAW, PSN and BN prepressed datasets. (i.e.,Top: batch1, Bottom: batch2. Left: raw data, Middle: PSN, and Right: BN).

Figure 2 show SERS spectrum profiles for *RAW*, and *PSN* and *BN* preprocessing techniques. First row of the Figure 2 shows SERS spectrum of *batch1* datasets while the second row of the Figure 2 shows *batch2* datasets. As *PSN* normalizes only the signal power, it only adjusts the range of values while preserving the shape of the spectrum. In contrast, *BN* transforms the signal shape by incorporating variance of the batch dataset and highlighting the discriminating features.

**Figure 3:**
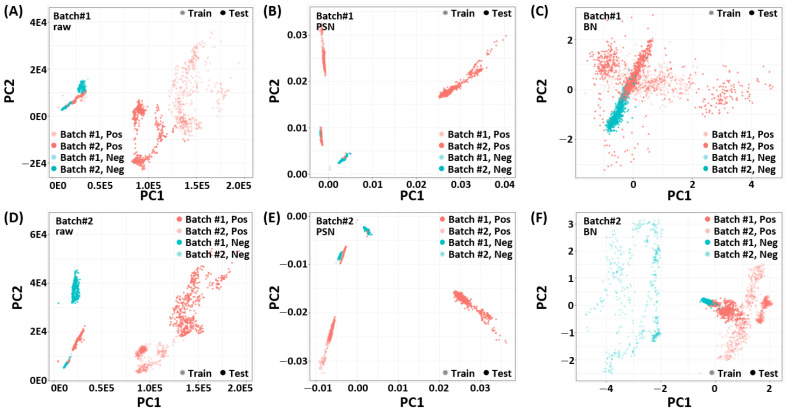
Visualization of the PCA embedding of R6G SERS Spectrum for *RAW* (**A**,**D**), power spectrum density normalization (PSN, **B**,**E**), and feature-specific batch normalization (BN, **C**,**F**) methods. The PCA embedding is learned with 80% training dataset of one batch and the dataset of the other batch is projected using the learned PCA embedding. Top: batch1, Bottom: batch2. Left: raw data, Middle: PSN, and Right: BN.

Before building and evaluating machine learning models, we analyze the effect of different preprocessing techniques. Towards this end, we visualized the preprocessed low-dimensional embedding of R6G. Figure 3 shows the PCA embedding of the SERS spectrum for each batch and class (positive/negative) of R6G according to the preprocessing methods. As shown in Figure 3A,D, respectively, positive and negative samples of *batch1* and *batch2* are clustered in the raw data in a way that they can not be linearly separated in their respective classes. Therefore, we cannot use a single classifier to separate positive and negative samples of both batches in the given raw data alone. This indicates that there exist some domain generalization problems which can seriously affect the performance of the classifier on unseen data/*batch*.

To handle the aforementioned *batch-effect*, we investigate two different preprocessing techniques explained in Section 2. Figure 3B,E, respectively, show the PCA embedding of *batch1* and *batch2* using PSN. Although the PSN showed better alignment between two batches, it did not remove the *batch-effect*. In contrast, the proposed BN shows desired *batch-effect* removal in Figure 3C,F and improves the class separability.

It is noteworthy to point out that the PCA is a linear embedding technique and it may not represent the actual class separability in nonlinear space (which is explored in MLP). However, it indicates the effect of preprocessing techniques and their importance for designing a reliable prediction model that can work for varying measurement conditions.

### 3.2. Performance Evaluation of SERSNet

The model is trained and tested for cross-batch datasets. We perform 10 independent trials and report mean and standard deviations for each performance metric. Table 3 shows the individual and average results of different batches. The results are shown for the MLP model that is trained using *RAW*, *PSN*, and *BN* preprocessing techniques. It can be seen from the results that the proposed method (*BN* + MLP) has consistent performance for both batches and it outperforms the *RAW* and *PSN* preprocessing techniques. Overall, the proposed method (*BN* + MLP) has achieved 0.969, 0.977, 0.930, 0.959, and 0.917 average accuracy, F1-Score, MCC, BACC and YI, respectively, which in turn are 0.310, 0.465, 0.929, 0.501, and 0.170, and 0.298, 0.206, 0.411, 0.210, and 0.083 units higher than the *RAW* and *PSN*-based implementations.

**Figure 4:**
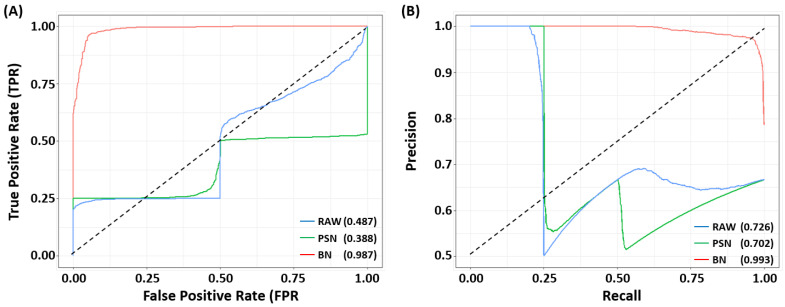
(**A**) ROC and (**B**) PR curves of *RAW* (blue), power spectrum density normalization (PSN, green), and feature-specific batch normalization (BN, red) methods + *SERSNet*. As anticipated the proposed (BN + MLP) SERSNet showed almost perfect ROC and PR curves while RAW showed relatively better curves than PSN case. The values in the parentheses are AUROC and AUPRC.

The aforementioned metrics are threshold dependent, therefore, to analyze threshold independent performance we plot the receiver operating characteristic (ROC), and precision–recall curves (PRC) and calculated their area under the curve (AUC). To summarize the statistics, the curves are drawn by taking the average of the results for both batches. Figure 4A shows the ROC curve of SERSNet for independent test sets using *RAW*, *PSN*, and *BN* datasets. As expected, the proposed model showed almost perfect ROC curves and the area under the ROC curve (AUROC) of the proposed model is 0.987. In contrast, the curves for the RAW and BN cases are below the random-chance line (AUROC = 0.5) and have AUROC 0.487 and 0.388, respectively. Similarly, Figure 4B shows the precision–recall curve of SERSNet for the same configuration. Again the proposed model has shown almost perfect PR curves and the area under the PR curve (AUPRC) of the proposed model is 0.993. RAW dataset showed relatively better performance than PSN case (0.726 vs. 0.702). From this analysis, we confirmed that the proposed *BN* + SERSNet have robust performance across the wide range of threshold values.

### 3.3. Comparative Analysis

For comparative analysis, we consider *logistic regression* (LR) with ridge constraint (with $\ell_{2}$ penalty of C=1), *Gaussian Naive Bayes* (NB) [36] with prior of (0.5 and 0.5), *decision tree* (DT) [37] with ’Gini’ as measure of impurity, *random forest* (RF) with 100 estimators, support vector machine with a *linear kernel* (LinSVM) [38,39], and with *radial basis function kernel* (RBFSVM) [40]. We use balanced class weights and $\ell_{2}$ penalty of C=1 for both SVM models, and consider kernel coefficient $\gamma =1/(2000\times \sigma_{s_{i}}^{2})$ for the RBFSVM. Here, $\sigma_{s_{i}}^{2}$ stands for variance of the spectrum.

All models are implemented using *scikit-learn* package [35] on Python 3 and are trained and tested using a bath-normalized dataset as it provides the best tolerate against domain adaptation problems (as shown in Table 3). The models are trained and tested using cross-batch and same-batch datasets for inter-batch and intra-batch performance analysis respectively. All experiments are repeated for 10 independent trials and mean and standard deviations of performance statistics are reported.

#### 3.3.1. Inter-Batch Prediction Performance

In this study, we analyze the cross-batch training performance of each model. In particular, we compare the balanced accuracy of the proposed model with the aforementioned machine learning models. As shown in Table 4, for *batch1* training and *batch2* testing case, LR and LinSVM, show similar performance as compared to the proposed model. However, for *batch2* training and *batch1* testing case, LR and LinSVM show the worst performance among all other models, and only the proposed model has achieved satisfactory performance (BACC 0.960). As these two models are linear and all the nonlinear models showed relatively better performance in the *batch2* training and *batch1* testing case, it is most likely that the classification boundary is highly nonlinear. Since the proposed model can learn nonlinear classification boundaries more efficiently than other models, it renders the best performance among all other models. In a nutshell, the proposed method shows consistent performance for both batches and achieve 0.959 BACC that is 0.256, 0.283, 0.209, 0.274, 0.478, and 0.238 units higher than the LR, LinSVM, NB, DT, RF, and RBFSVM-based implementations, respectively.

We also observed that the model trained on the *batch1* dataset performed better than the model trained on the *batch2* dataset. All models except the proposed one do not work well in the *batch2* dataset training *batch1* data test scenario. That may be due to the low probability of separation between positive and negative samples in the *batch2* data set, especially the 0.0 μM and 0.1 μM samples, as shown in Figure 2. Therefore, to classify datasets with low separability, it is recommended to train the classifier on a dataset of a high dynamic range that can better differentiate between positive and negative examples.

#### 3.3.2. Intra-Batch Prediction Performance

In addition to inter-batch analysis, i.e., different-batch training and testing, we also analyze the performance of individual models within each batch using 10-folds cross-validation, as shown in Table 5. As inter-batch classification is trivial as expected, we found that almost all models perform equally well in this scenario. The simplest and linear models perform best whereas the proposed model (MLP + *BN*) performs second-best achieving a BACC of 0.997±0.006 which is only 0.001 units lower than the LR model. On the other hand, the NB performs the worst while the Tree-based models such as the DT and RF perform similar to the proposed model. We argue that without defining evaluation protocol (intra-batch or inter-batch) reporting high performance may be misleading. These results indicate that the intra-batch analysis is a trivial task, and it can be decisive in selecting the best model. One of the main contributions of this study is that we highlight this reporting issue and provide an evaluation protocol for the machine learning-based SERS classification models.

## 4. Conclusions

In this study, an optimal preprocessing technique, model training, and evaluation method for the SERS-based R6G molecule detection were proposed, and a benchmark dataset was provided to lay the foundation for advanced model construction. The proposed model showed excellent performance on the R6G molecule detection task compared to other machine learning models. In this study, we considered R6G as a proof-of-concept molecule for the basic SERS + DL biosensing concept, which is widely used for the characterization of SERS-based biosensors. Based on the model developed in this study, we plan to conduct applied research on various biomolecules such as proteins and bacterial cell detection in the future. Our model can be applied to these applications to improve the reproducibility of SERS-based biosensors, as evident in the present study. As intra-batch analysis is a trivial task, we argue that without defining evaluation protocol (intra-batch or inter-batch) reporting high performance can be misleading. One of the main contributions of this study is that we highlight this reporting issue and provide an evaluation protocol and a public dataset for the machine learning-based SERS classification models. We believe that these results can be used as a benchmark for the further development of advanced biomolecule detection models based on SERS measurements, such as end-to-end deep learning models.

## Figures and Tables

**Table 1:** Summary of research conducted on machine learning-based SERS biosensor.

Problem	Technique	Reference
Cell type classification	SVM	[7] (2018)
Origin of milk classification (from 4 species)	RF	[11] (2018)
Blood origin classification	PLSDA	[14] (2018)
Illicit drug detection	SVM	[13] (2018)
Prenatal disease diagnosis	PCA-SVM	[15] (2018)
Food colorants detection	PCA	[12] (2018)
Prostate cancer detection	PCA	[17] (2018)
Odor source direction identification	SVM, CNN	[16] (2019)
DNA sensing	SVM	[6] (2020)
Drug recognition in urine	FCNN, CNN	[19] (2020)
Wine flavor classification	SVM	[20] (2021)
Pathogen detection	DNN	[21] (2021)

**Table 2:** Sample Statistics of the R6G.

	Negative		Positive			
concentration (μM)	0	0.01	0.1	10	100	10,000
batch1	500	0	0	500	0	500
batch2	0	500	500	0	500	0

**Table 3:** Performance summary of the SERSNet.

Model	Train	Test	Accuracy	Sensitivity	Specificity	F1-Score	MCC	BACC	Youden’s Index
RAW	Batch1	Batch2	0.667 ± 0.000	1.000 ± 0.000	0.000 ± 0.000	0.800 ± 0.000	0.000 ± 0.000	0.500 ± 0.000	0.000 ± 0.000
	Batch2	Batch1	0.651 ± 0.034	0.976 ± 0.050	0.000 ± 0.000	0.788 ± 0.025	−0.042 ± 0.088	0.488 ± 0.025	−0.024 ± 0.05
	Average		0.659 ± 0.025	0.988 ± 0.037	0.000 ± 0.000	0.794 ± 0.019	−0.021 ± 0.064	0.494 ± 0.018	−0.012 ± 0.037
PSN	Batch1	Batch2	0.676 ± 0.092	0.517 ± 0.141	0.995 ± 0.008	0.669 ± 0.129	0.510 ± 0.103	0.756 ± 0.068	0.511 ± 0.135
	Batch2	Batch1	0.667 ± 0.000	0.500 ± 0.000	1.000 ± 0.000	0.667 ± 0.000	0.500 ± 0.000	0.750 ± 0.000	0.500 ± 0.000
	Average		0.671 ± 0.064	0.508 ± 0.098	0.997 ± 0.006	0.668 ± 0.089	0.505 ± 0.071	0.753 ± 0.047	0.506 ± 0.093
Proposed (BN)	Batch1	Batch2	0.971 ± 0.002	0.999 ± 0.002	0.916 ± 0.007	0.979 ± 0.002	0.936 ± 0.005	0.957 ± 0.003	0.915 ± 0.007
	Batch2	Batch1	0.966 ± 0.006	0.979 ± 0.009	0.940 ± 0.011	0.975 ± 0.004	0.924 ± 0.013	0.960 ± 0.005	0.920 ± 0.011
	Average		0.969 ± 0.005	0.989 ± 0.012	0.928 ± 0.015	0.977 ± 0.004	0.930 ± 0.011	0.959 ± 0.005	0.917 ± 0.009

**Table 4:** Performance comparison between the proposed model and 6 ML models. The proposed model showed the best independent test balanced accuracy (BACC) results for both batch datasets.

Models	Train/Test		Average
	Batch1/Batch2	Batch2/Batch1	
**LR**	0.960 ± 0.000	0.446 ± 0.032	0.703 ± 0.257
**LinSVM**	0.953 ± 0.001	0.399 ± 0.010	0.676 ± 0.277
**NB**	0.749 ± 0.000	0.750 ± 0.000	0.750 ± 0.001
**DT**	0.737 ± 0.198	0.633 ± 0.119	0.685 ± 0.052
**RF**	0.431 ± 0.032	0.530 ± 0.007	0.481 ± 0.049
**RBFSVM**	0.894 ± 0.003	0.548 ± 0.004	0.721 ± 0.173
**Proposed**	0.957 ± 0.003	0.960 ± 0.005	0.959 ± 0.002

**Table 5:** Performance comparison between the proposed model and 6 ML models. The proposed model showed the best 10-fold CV test balanced accuracy (BACC) results for both batch datasets.

Model	Train/Test		Average
	Batch1/Batch1	Batch2/Batch2	
LR	0.999 ± 0.003	0.996 ± 0.007	0.998 ± 0.006
LinSVM	0.998 ± 0.004	0.997 ± 0.006	0.997 ± 0.005
NB	0.789 ± 0.024	0.789 ± 0.030	0.789 ± 0.026
DT	0.979 ± 0.020	0.946 ± 0.020	0.962 ± 0.026
RF	0.994 ± 0.005	0.980 ± 0.012	0.987 ± 0.012
RBFSVM	0.998 ± 0.003	0.974 ± 0.017	0.986 ± 0.017
Proposed	0.998 ± 0.003	0.995 ± 0.007	0.997 ± 0.006

## Data Availability

The code and the dataset utilized in this work are available at the Author’s GitHub (https://github.com/psychemistz/sersnet, accessed on 30 November 2021).

## References

[1] Langer J., Jimenez de Aberasturi D., Aizpurua J., Alvarez-Puebla R.A., Auguié B., Baumberg J.J., Bazan G.C., Bell S.E., Boisen A., Brolo A.G. (2019). Present and future of surface-enhanced Raman scattering. ACS Nano.

[2] Zhao X., Li M., Xu Z. (2018). Detection of foodborne pathogens by surface enhanced raman spectroscopy. Front. Microbiol..

[3] Bodelón G., Pastoriza-Santos I. (2020). Recent progress in surface-enhanced Raman scattering for the detection of chemical contaminants in water. Front. Chem..

[4] Shaban M., Galaly A. (2016). Highly sensitive and selective in-situ SERS detection of Pb 2+, Hg 2+, and Cd 2+ using nanoporous membrane functionalized with CNTs. Sci. Rep..

[5] Zhou Y., Zhao G., Bian J., Tian X., Cheng X., Wang H., Chen H. (2020). Multiplexed SERS barcodes for anti-counterfeiting. ACS Appl. Mater. Interfaces.

[6] Meyer N., Janot J.M., Lepoitevin M., Smietana M., Vasseur J.J., Torrent J., Balme S. (2020). Machine learning to improve the sensing of biomolecules by conical track-etched nanopore. Biosensors.

[7] Hassoun M., Rüger J., Kirchberger-Tolstik T., Schie I.W., Henkel T., Weber K., Cialla-May D., Krafft C., Popp J. (2018). A droplet-based microfluidic chip as a platform for leukemia cell lysate identification using surface-enhanced Raman scattering. Anal. Bioanal. Chem..

[8] Singh A., Sharma A., Ahmed A., Sundramoorthy A.K., Furukawa H., Arya S., Khosla A. (2021). Recent Advances in Electrochemical Biosensors: Applications, Challenges, and Future Scope. Biosensors.

[9] Peng Y., Lin C., Long L., Masaki T., Tang M., Yang L., Liu J., Huang Z., Li Z., Luo X. (2021). Charge-transfer resonance and electromagnetic enhancement synergistically enabling MXenes with excellent SERS sensitivity for SARS-CoV-2 S protein detection. Nano-Micro Lett..

[10] Yang Y., Peng Y., Lin C., Long L., Hu J., He J., Zeng H., Huang Z., Li Z.Y., Tanemura M. (2021). Human ACE2-Functionalized Gold “Virus-Trap” Nanostructures for Accurate Capture of SARS-CoV-2 and Single-Virus SERS Detection. Nano-Micro Lett..

[11] Amjad A., Ullah R., Khan S., Bilal M., Khan A. (2018). Raman spectroscopy based analysis of milk using random forest classification. Vib. Spectrosc..

[12] Ai Y.J., Liang P., Wu Y.X., Dong Q.M., Li J.B., Bai Y., Xu B.J., Yu Z., Ni D. (2018). Rapid qualitative and quantitative determination of food colorants by both Raman spectra and Surface-enhanced Raman Scattering (SERS). Food Chem..

[13] Dies H., Raveendran J., Escobedo C., Docoslis A. (2018). Rapid identification and quantification of illicit drugs on nanodendritic surface-enhanced Raman scattering substrates. Sens. Actuators B Chem..

[14] Doty K.C., Lednev I.K. (2018). Differentiation of human blood from animal blood using Raman spectroscopy: A survey of forensically relevant species. Forensic Sci. Int..

[15] Kim W., Lee S.H., Kim J.H., Ahn Y.J., Kim Y.H., Yu J.S., Choi S. (2018). Based surface-enhanced Raman spectroscopy for diagnosing prenatal diseases in women. ACS Nano.

[16] Thrift W.J., Cabuslay A., Laird A.B., Ranjbar S., Hochbaum A.I., Ragan R. (2019). Surface-enhanced Raman scattering-based odor compass: Locating multiple chemical sources and pathogens. ACS Sens..

[17] Lee W., Nanou A., Rikkert L., Coumans F.A., Otto C., Terstappen L.W., Offerhaus H.L. (2018). Label-free prostate cancer detection by characterization of extracellular vesicles using raman spectroscopy. Anal. Chem..

[18] Lussier F., Thibault V., Charron B., Wallace G.Q., Masson J.F. (2020). Deep learning and artificial intelligence methods for Raman and surface-enhanced Raman scattering. TrAC Trends Anal. Chem..

[19] Weng S., Yuan H., Zhang X., Li P., Zheng L., Zhao J., Huang L. (2020). Deep learning networks for the recognition and quantitation of surface-enhanced Raman spectroscopy. Analyst.

[20] Leong Y.X., Lee Y.H., Koh C.S.L., Phan-Quang G.C., Han X., Phang I.Y., Ling X.Y. (2021). Surface-Enhanced Raman Scattering (SERS) Taster: A Machine-Learning-Driven Multireceptor Platform for Multiplex Profiling of Wine Flavors. Nano Lett..

[21] Ciloglu F.U., Caliskan A., Saridag A.M., Kilic I.H., Tokmakci M., Kahraman M., Aydin O. (2021). Drug-resistant Staphylococcus aureus bacteria detection by combining surface-enhanced Raman spectroscopy (SERS) and deep learning techniques. Sci. Rep..

[22] Jinadasa M.W.N., Kahawalage A.C., Halstensen M., Skeie N.O., Jens K.J. (2019). Deep Learning Approach for Raman Spectroscopy. Recent Developments in Atomic Force Microscopy and Raman Spectroscopy for Materials Characterization.

[23] Wang C., Xiao L., Dai C., Nguyen A.H., Littlepage L.E., Schultz Z.D., Li J. (2020). A Statistical Approach of Background Removal and Spectrum Identification for SERS Data. Sci. Rep..

[24] Cialla D., Pollok S., Steinbrücker C., Weber K., Popp J. (2014). SERS-based detection of biomolecules. Nanophotonics.

[25] Chan T.Y., Liu T.Y., Wang K.S., Tsai K.T., Chen Z.X., Chang Y.C., Tseng Y.Q., Wang C.H., Wang J.K., Wang Y.L. (2017). SERS detection of biomolecules by highly sensitive and reproducible Raman-enhancing nanoparticle array. Nanoscale Res. Lett..

[26] Wu L., Wang W., Zhang W., Su H., Liu Q., Gu J., Deng T., Zhang D. (2018). Highly sensitive, reproducible and uniform SERS substrates with a high density of three-dimensionally distributed hotspots: Gyroid-structured Au periodic metallic materials. NPG Asia Mater..

[27] Cong S., Wang Z., Gong W., Chen Z., Lu W., Lombardi J.R., Zhao Z. (2019). Electrochromic semiconductors as colorimetric SERS substrates with high reproducibility and renewability. Nat. Commun..

[28] Chen K.H., Pan M.J., Jargalsaikhan Z., Ishdorj T.O., Tseng F.G. (2020). Development of Surface-Enhanced Raman Scattering (SERS)-Based Surface-Corrugated Nanopillars for Biomolecular Detection of Colorectal Cancer. Biosensors.

[29] Tzeng Y., Lin B.Y. (2020). Silver SERS adenine sensors with a very low detection limit. Biosensors.

[30] Vikulina A.S., Stetsyura I.Y., Onses M.S., Yilmaz E., Skirtach A.G., Volodkin D. (2021). Mesoporous One-Component Gold Microshells as 3D SERS Substrates. Biosensors.

[31] Sung C.J., Chao S.H., Hsu S.C. (2021). Rapid Detection of Glucose on Nanostructured Gold Film Biosensor by Surface-Enhanced Raman Spectroscopy. Biosensors.

[32] Deb S.K., Davis B., Knudsen G.M., Gudihal R., Ben-Amotz D., Davisson V.J. (2008). Detection and relative quantification of proteins by surface enhanced Raman using isotopic labels. J. Am. Chem. Soc..

[33] Liu Y.C., Yu C.C., Sheu S.F. (2006). Low concentration rhodamine 6G observed by surface-enhanced Raman scattering on optimally electrochemically roughened silver substrates. J. Mater. Chem..

[34] Veselkov K.A., Vingara L.K., Masson P., Robinette S.L., Want E., Li J.V., Barton R.H., Boursier-Neyret C., Walther B., Ebbels T.M. (2011). Optimized preprocessing of ultra-performance liquid chromatography/mass spectrometry urinary metabolic profiles for improved information recovery. Anal. Chem..

[35] Pedregosa F., Varoquaux G., Gramfort A., Michel V., Thirion B., Grisel O., Blondel M., Prettenhofer P., Weiss R., Dubourg V. (2011). Scikit-learn: Machine learning in Python. J. Mach. Learn. Res..

[36] Rennie J.D., Shih L., Teevan J., Karger D.R. Tackling the poor assumptions of naive bayes text classifiers. Proceedings of the 20th International Conference on Machine Learning (ICML-03).

[37] Quinlan J.R. (1986). Induction of decision trees. Mach. Learn..

[38] Chang C.C., Lin C.J. (2011). LIBSVM: A library for support vector machines. ACM Trans. Intell. Syst. Technol. (TIST).

[39] Fan R.E., Chang K.W., Hsieh C.J., Wang X.R., Lin C.J. (2008). LIBLINEAR: A library for large linear classification. J. Mach. Learn. Res..

[40] Wang J., Chen Q., Chen Y. (2004). RBF kernel based support vector machine with universal approximation and its application. International Symposium on Neural Networks.

